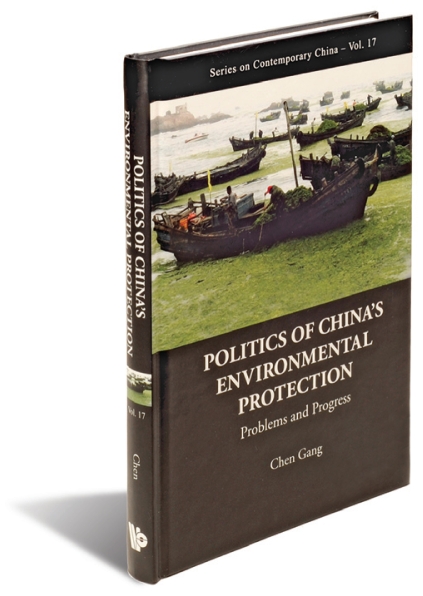# Politics of China’s Environmental Protection: Problems and Progress

**Published:** 2010-04

**Authors:** Changhua Wu

**Affiliations:** *Changhua Wu is the Greater China Director of The Climate Group, with a focus on forging public and private partnership with technology solution providers, financial institutions, and city and regional governments to scale up low-carbon solutions in China.*

At the recent Copenhagen climate conference, the United States pushed transparency and accountability to such a high level of importance that it would not commit to aggressive emissions reduction without a commitment from large developing countries, particularly China, to take dramatic steps to reduce emissions.

International politics played a big role in this complex debate and negotiation. China has had reasons to fight for sovereignty and right of development in the context of a global climate regime based on “common but differentiated” responsibilities and the Bali Action Plan, and China does need to improve its environmental governance through continued political and bureaucratic system reforms. As Chen Gang rightly articulates in *Politics of China’s Environmental Protection*, “ecological protection is not only an issue of environmental engineering, technology, and economics, but also a tough political task that has to resort to the improvement of governance and policy adjustment.”

This argument is not new among China’s environmental policy analysts. Many scholars, Chinese and foreign, have concluded that what keeps China from effectively enforcing those ambitious legislation and policy targets is the existing political and bureaucratic systems or environmental governance mechanisms. What comes first—economic growth or environmental protection? This question challenges conventional practices and wisdom in industrialized countries, which achieved industrialization and urbanization without constraints of carbon or environmental carrying capacity. China so far has not managed to shift its development paradigm based on sustainability.

What to do, then? Environmental protection, to a large extent, cannot be addressed by a single government agency, as the author notes in “Bureaucratic Structures on Environmental Protection” (Chapter 2). Rather, it requires coordination to ensure that environmental protection is not sacrificed or compromised by other economic growth priorities. Such coordination must extend to regional and local levels, among provinces and among river basins. For local leaders, economic growth versus environmental protection is also a challenge in decision making. When local economic growth is the dominating performance indicator, and if no mechanism holds local leaders accountable for environmental protection, national laws and regulations and policy targets would remain ineffective and unenforced.

As described in Chapters 3 and 4, the Ministry of Environmental Protection in China was strengthened recently when granted a Cabinet seat. The Ministry has also started to address the coordination matter by setting up six regional offices to strengthen enforcement. It is a huge learning process, and we must wait and see how effective it will be. Both nationally and locally, the environmental agency remains a relatively weak government arm, often ignored in favor of national economic growth. The author describes how these policy issues relate to air and water pollution and climate change, and presents several illustrative case studies.

China has started to invest heavily in environmental protection, with 1–2% of its gross domestic product now devoted to protecting its environment. But much of the investment has gone to large projects, as illustrated by the author’s case studies, such as the “Taihu Algae Crisis” (Chapter 7). Such projects have merit, but environmental protection is a long-term cause that requires institutional infrastructure and programs to ensure that economic growth decisions will not compromise environmental quality.

Climate change (Chapter 9) is another complicated and relatively new matter for China that requires more effort and learning. China’s legislative framework is not clearly defined to address greenhouse gas emissions. Premier Wen Jiabao chairs the group to coordinate work among government agencies on climate change, which is limited to certain key issues and cannot address all conflicts among agencies. The National Development and Reform Commission, the agency in charge of planning and coordination, could make conflicting policy that would slow the process toward low carbon development.

This book is a good addition to the Contemporary China series, offering another perspective on the challenges China faces in balancing economic development and environmental quality. China’s past record is not sustainable, and an alternative paradigm is a must. The book shows clearly the difficulties of the world’s most populous country in achieving this balance. With 1.3 billion people to feed, shelter, and transport, China faces a serious challenge.

What is encouraging is the emerging alignment of clear vision, strong policy support, technology innovation, capital flows, and deployment of technologies and market in China that offer the biggest opportunity to integrate economic growth with environmental protection. As many Western leaders argued in Copenhagen, even if all the industrialized nations achieved zero carbon emissions today, we still cannot tackle the climate change challenge if such large emerging economies as China continue on its current path. China’s success or failure to achieve a low-carbon future has huge repercussions for our global future. The challenge for established industrialized countries, as set out in this book, is to work with China constructively, share expertise and experience, offer resources and stop finger pointing. We no longer have time and resources to waste.

## Figures and Tables

**Figure:**